# Alkali Halide Aqueous Solutions Under Pressure: A Non-Equilibrium Molecular Dynamics Investigation of Thermal Transport and Thermodiffusion

**DOI:** 10.3390/e27020193

**Published:** 2025-02-13

**Authors:** Guansen Zhao, Fernando Bresme

**Affiliations:** Department of Chemistry, Molecular Sciences Research Hub Imperial College, London W12 0BZ, UK; g.zhao21@imperial.ac.uk

**Keywords:** thermodoffusion, alkali halide solutions, GPa pressure, thermal conductivity, water

## Abstract

Thermal gradients induce thermodiffusion in aqueous solutions, a non-equilibrium effect arising from the coupling of thermal and mass fluxes. While thermal transport processes have garnered significant attention under standard conditions, thermal transport at high pressures and temperatures, typical of the Earth’s crust, has escaped scrutiny. Non-equilibrium thermodynamics theory and non-equilibrium molecular dynamics simulations provide an excellent means to quantify thermal transport under extreme conditions and establish a connection between the behaviour of the solutions and their microscopic structure. Here, we investigate the thermal conductivity and thermal diffusion of NaCl and LiCl solutions in the GPa pressure regime, targeting temperatures between 300 K and 1000 K at 1 molal concentration. We employ non-equilibrium molecular dynamics simulations along with the Madrid-2019 and TIP4P/2005 force fields. The thermal conductivity of the solutions increases significantly with pressure, and following the behaviour observed at standard pressure, the thermal conductivity is lower than that of pure water. The reduction in thermal conductivity is significant in the GPa pressure regime, ∼3% for 1 molal NaCl and LiCl solutions. We demonstrate that under GPa pressure conditions, the solutions feature thermophobic behaviour, with ions migrating towards colder regions. The pronounced impact of pressure is more evident in LiCl solutions, which display a thermophilic to thermophobic “transition” at pressures above 0.25 GPa. We discuss a correlation between the solution’s thermophobicity and the disruption of the water hydrogen bond structure at high pressure, where the water structure resembles that observed in simple liquids.

## 1. Introduction

Electrolyte solutions experience high pressures of approximately 0.1 to 1 GPa in the Earth’s mantle [1,2] and in the deep oceanic trenches or extraterrestrial oceans beneath thick ice crusts [3]. Previous studies have explained how compression affects the rates of physicochemical [4] and biological [5] processes. Experimental [6,7] and computational [8,9] investigations have demonstrated that high pressure in the GPa range considerably modifies the hydrogen bonding network of liquid water, resulting in a structure reminiscent of that observed in simple liquids. The alteration of the hydrogen-bond network influences the thermal transport properties of water, affecting the energy transfer efficiency between molecules [10,11].

The Ludwig-Soret effect, discovered in the late 19th century, is a non-equilibrium coupling effect between heat and mass fluxes [12,13]. It has been demonstrated that applying a thermal gradient to an aqueous electrolyte solution induces a concentration gradient. This coupling effect between heat and mass fluxes can be elucidated at the macroscopic scale using linear non-equilibrium thermodynamics theory and the corresponding phenomenological equations, which quantify the extent of mass separation in response to the thermal gradient [14]. The tendency of solutes to migrate towards either hot (thermophilic) or cold (thermophobic) regions in the thermal field is believed to influence, for example, the thermophoretic response of colloidal suspensions [15,16]. Furthermore, the Soret-Ludwig phenomenon provides a theoretical foundation for the design of analytical devices to monitor the thermophoretic motion of biomolecules [17,18].

The Soret coefficient $s_{T}=D_{T}/D$ is defined by the ratio of the thermal diffusion coefficient ($D_{T}$) and the inter-diffusion coefficient (*D*), and it is often employed to quantify the magnitude of the thermodiffusion effect. The sign change and inversion point of the Soret coefficient, where $s_{T}=0$, have been reported in experiments involving liquid mixtures and electrolyte aqueous solutions. At the inversion point, the Soret coefficient changes from thermophilic ($s_{T}<0$) to thermophobic ($s_{T}>0$) as the temperature increases [15,19]. Molecular dynamics simulations of alkali halide solutions reproduced the experimental behaviour using various forcefields [10,20,21,22]. Moreover, minima in the Soret coefficient have been reported experimentally and in simulations for different electrolyte solutions [23,24,25,26]. The simulations highlighted a correlation between the thermodiffusion response and the minimum in the Soret coefficient. Furthermore, recent studies of electrolyte solutions at standard pressure conditions demonstrated a correlation between thermophobicity and the disruption of the oxygen-oxygen structural characteristics of water [21]. This is an aspect we aim to address in this work, since the structure of water is significantly modified at high pressure, and we expect, based on previous observations, an enhancement of the thermophobic response. The predominance of thermophobic response is relevant to the behaviour of solutions in, for example, oceans or the Earth’s mantle since heat-mass coupling would lead to a preferential accumulation of salt in colder regions.

The impact of pressure on ionic solutions has garnered significant interest, as high pressure and temperature conditions can be found in the Earth’s crust. Several studies have focused on the structure of ion solutions, electric conductivity, solution dielectric permittivity, and the properties of pure water at pressures in the GPa range [27,28,29,30,31,32,33,34,35]. Metamorphic fluids contain water as one major component, and understanding the properties of water, such as mass transport within the Earth, is of particular interest. There is evidence that the properties of these fluids differ considerably from those of fluids under standard conditions [36,37]. The properties of high-pressure fluids are also of interest in other bodies within our solar system, such as Europa, where oceans have been proposed with pressures reaching the 0.1 GPa range [3].

In this work, we investigate the impact of pressure on the thermal transport properties of NaCl and LiCl solutions. NaCl is a significant component of sea salt, and we have chosen a concentration of 1 m close to that found in the ocean. The lithium ion is relevant in energy applications, and it exhibits stronger thermophilicity than other alkali cations, such as ${\mathrm{Na}}^{+}$ [20,23,24,38]. Also, alkali chlorides are major components of deep crustal fluids [37]. We report both Soret coefficients and thermal conductivities for these salts, analyze the water structure and hydration shells of ions at high pressure and discuss their impact on thermal transport and thermal diffusion. These thermophysical properties of water and its solutions at high pressure might be of interest to inform thermodynamic models of the upper mantle [36] since water plays a significant role in the heat and mass transport inside the Earth [37].

We employ the TIP4P/2005 water [39] and Madrid-2019 forcefields [40] to simulate water molecules and ions, respectively. TIP4P/2005 is one of the most accurate non-polarizable rigid water models, widely used to study supercooled water and ice polymorphs [41,42,43]. The Madrid-2019 forcefield is designed to conduct simulations with TIP4P/2005 water, utilising scaled charges to replicate the thermophysical and structural properties of electrolyte solutions [40]. A recent investigation demonstrated that the Madrid-2019 model accurately reproduces the dependence of thermal conductivity and thermal diffusion of NaCl and LiCl under standard pressure conditions [21].

## 2. Materials and Methods

**Figure 1:**
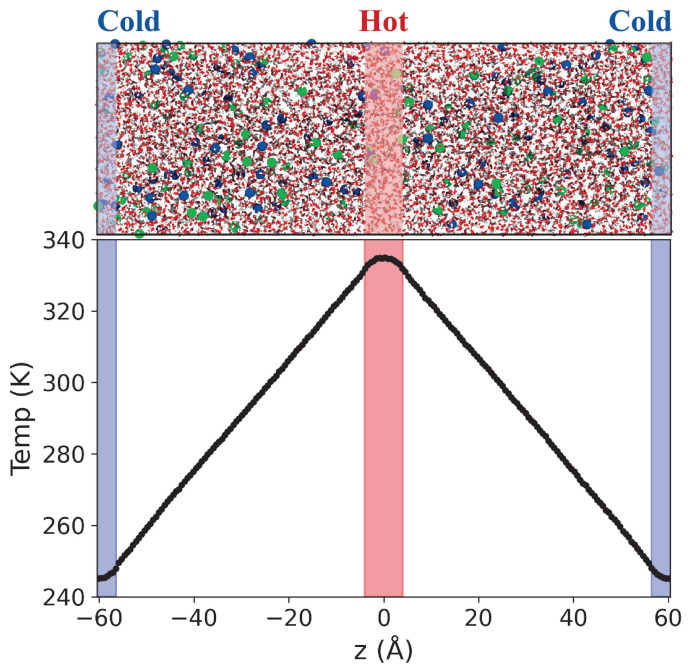
(**Top**) Snapshot of the simulations cell employed in the NEMD simulation of 1 molal LiCl aqueous solution. The red, white, blue and green spheres represent the oxygen, hydrogen atoms, and ${\mathrm{Li}}^{+}$ and ${\mathrm{Cl}}^{-}$ ions, respectively. The thermostatting regions are highlighted in red (hot, center of the box) and blue (cold, box edges). (**Bottom**) Temperature profile obtained with our NEMD simulation method.

To compute the thermal conductivity and Soret coefficient of the electrolyte solutions, we employed boundary-driven non-equilibrium molecular dynamics (NEMD) simulations [44]. All NEMD simulations were performed using LAMMPS (vs. 3 Mar 2020) [45] with a 1 fs timestep. This approach involves thermostatting the solutions in specific regions, located at the centre and edges of the simulation box, to predefined hot ($T_{H}$) and cold ($T_{C}$) temperatures (see Figure 1). The thermostat regions had a thickness of 8 Å, and the temperature of the molecules and ions were reset every timestep using the Langevin thermostat [46,47] with a coupling constant of 500 fs. The total momentum of the simulation box was reset every timestep. The electrostatic interactions and long-range dispersion interactions were calculated using the particle-particle particle-mesh (PPPM) method [48] using a force accuracy of 5 × $10^{-5}$ kcal/(mol Å) for the coulombic interactions, and $10^{-4}$ and 2 × $10^{-4}$ kcal/(mol Å) for the real and k-space contributions of the dispersion interactions, respectively. In setting the thermal gradients, we followed the approach discussed in reference [43], where it was shown that thermal gradients similar to those employed here result in a linear response.

We used cuboid simulation boxes with a size ratio $\left\{L_{x}:L_{y}:L_{z}\right\}=\left\{1:1:3\right\}$, and $L_{x}\approx 40$ Å. The heat flux was set up along the *z* direction. A typical simulation box contained 6663 TIP4P/2005 water molecules [39] and 120 ion pairs corresponding to a 1 mol/kg concentration. We used the Madrid-2019 forcefield [40] to describe the ion-ion and ion-water interactions. The system was pre-equilibrated for 5 ns using the isothermal-isobaric (NPT) ensemble at the target pressure and a temperature equal to the average of $T_{hot}$ and $T_{cold}$. Then, the average density during the NPT simulation was calculated, and the simulation cell vectors were set to the average values obtained from the NPT simulation. The thermostats were applied, and the NEMD simulations were performed at constant volume conditions. Following equilibration over 10 ns, the system reached the stationary state, and we calculated the local temperature and concentration profiles, averaging over at least 50 ns production run and 10 replicas, providing an accumulated sampling time of 0.5 μs. The thermal conductivity and the Soret coefficient were obtained from the analysis of the temperature and concentration profiles.

The thermal conductivity, λ(z), was determined using Fourier’s law by calculating the local thermal gradient ∇T(z), and the local temperature profile in the *z* direction,

(1) $$\lambda \left(z\right)=-\frac{J_{q}}{\nabla T(z)},$$

where $J_{q}$ denotes the heat flux in the direction of the thermal gradient. The heat flux was calculated using the continuity equation:

(2) $$J_{q}=\frac{\dot{Q}}{2A}.$$

**Figure 2:**
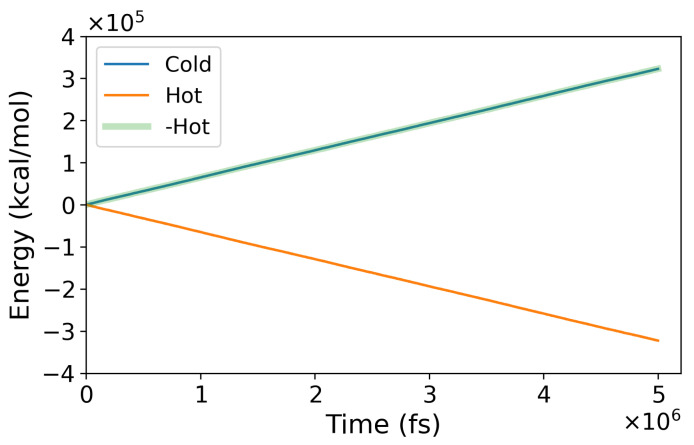
Energy exchanged at the hot and cold thermostats during a representative NEMD run. The blue and orange lines indicate the energy exchange in the cold and hot regions. The negative of the energy exchanged at the hot thermostats has been plotted in green for an easier comparison and test of energy conservation.

Here, $\dot{Q}$ represents the heat rate; *A* is the simulation box’s cross-sectional area perpendicular to the heat flux direction, and the factor “2” accounts for the two heat fluxes within the simulation cell. The heat rate was computed from the energy exchanged at the hot and cold thermostats, which should be equal and of opposite sign (see Figure 2). Our method provides excellent energy conservation, with the energy exchanged at the cold and hot thermostats being the same within computational accuracy.

For the Soret coefficient investigation, we considered the linear non-equilibrium formalism. The entropy production (σ) of a binary system based on the non-equilibrium thermodynamics [14,49]:

(3) $$\sigma =-\frac{1}{T^{2}}J_{q}\cdot \nabla T-\frac{1}{T}\sum_{i=1}^{2}J_{i}\cdot \nabla_{T}\mu_{i},$$

where $J_{q}$ and $J_{i}$ are the heat and mass fluxes, respectively; $\nabla_{T}\mu_{i}$ represents the chemical potential gradient of the *i*th component at constant temperature. In Equation (3), we have ignored charge fluxes since we did not consider external electrostatic fields in the simulations reported below.

Considering $\sum_{i}J_{i}=0$, we can write the phenomenological linear equations [14,21]:

(4) $$J_{q}=-L_{qq}\frac{\nabla T}{T^{2}}-\frac{L_{q1}}{T}\nabla_{T}\left(\mu_{1}-\mu_{2}\right),$$

(5) $$J_{1}=-L_{1q}\frac{\nabla T}{T^{2}}-\frac{L_{11}}{T}\nabla_{T}\left(\mu_{1}-\mu_{2}\right),$$

where $L_{\alpha \beta }$ are the Onsager coupling coefficients. When the system reaches the stationary state $J_{1}=0$, and Equation (5) can be rewritten as,

(6) $$\frac{\nabla_{T}\left(\mu_{1}-\mu_{2}\right)}{\nabla T}=-\frac{1}{T}\frac{L_{1q}}{L_{11}}=s_{T}\frac{d\mu_{1}}{d\ln w_{1}},$$

where $w_{1}$ is the solute weight fraction and $s_{T}$ is the Soret coefficient. Then the Soret coefficient can be obtained [14],

(7) $$s_{T}=-\frac{1}{T}{\left(\frac{\nabla \ln(w_{1}/w_{2})}{\nabla \ln T}\right)}_{J_{1}=0}=-\frac{1}{x_{1}x_{2}}{\left(\frac{\nabla x_{1}}{\nabla T}\right)}_{J_{1}=0}=-\frac{1}{b}{\left(\frac{db}{dT}\right)}_{J_{1}=0}.$$

In the equation above, $x_{1}$ and $x_{2}$ represent the molar fractions of solute and water, respectively; *b* is the solute molality. The derivation of the right hand side of Equation (7) can be found in ref. [21].

We also conducted equilibrium NPT simulations with Gromacs (vs. March 2021 [50]) to investigate the structural properties and the speed of sound of the solutions. The equilibrium simulations included 926 water molecules and 17 ion pairs, and the production runs were performed for at least 60 ns with a 1 fs timestep. Using the NPT simulation trajectories, we calculated the radial distribution functions (RDF) and the ion-water coordination numbers. The code TRAVIS [51,52] was employed to compute the spatial 3D density distribution functions (SDF) [53] of hydrogen and oxygen atoms in water. Additionally, we performed the mean square displacement analysis to calculate the diffusion coefficient of ions using the Einstein relation [54].

The fluctuation properties including isobaric thermal expansion coefficient (α) and isothermal compressibility ($k_{T}$) were computed employing the fluctuation relations [55] ${\langle \delta V\delta H\rangle }_{NPT}=k_{B}T^{2}V\alpha$, and ${\langle \delta V^{2}\rangle }_{NPT}=Vk_{B}Tk_{T}$, respectively. In the above equations, *V* is the volume; *H* is the enthalpy, $k_{B}$ is the Boltzmann constant; and $\delta P=P-P_{ens}$ where “ens” represents the ensemble average of property P. The speed of sound was obtained from:

(8) $$c_{s}=\sqrt{\frac{C_{P}}{C_{V}}\frac{1}{\rho k_{T}}},$$

where ρ is the mass density; $C_{P}$ and $C_{V}$ denote the isobaric and isochoric heat capacities, respectively.

## 3. Results

**Figure A1:**
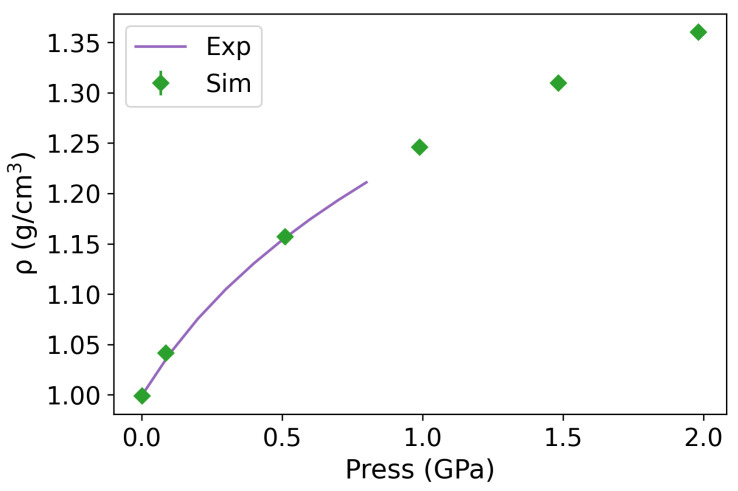
Equation of state from equilibrium NPT simulations (green points) at 10^−4^, 0.1, 0.5, 1, 1.5, and 2 GPa pressures and 290 K temperature. The simulation results are compared with experimental data (purple curve) from 10^−4^ to 0.8 GPa [60].

To evaluate the performance of the forcefield under high pressure, we computed the equation of state of water for the 290 K isotherm. The agreement between the simulated and experimental results up to 0.8 GPa is excellent (see Figure A1 in the Appendix A). Moreover, the equations of states obtained from NEMD simulations at standard conditions (see Table A2) agree with previous reports for the same force field using small samples and equilibrium conditions (see refs. [41,43]). It is important to note that TIP4P/2005 exhibits a shift in temperature with respect to the experimental coexistence diagram, and the liquid phase remains stable at pressures exceeding 0.8 GPa.

**Figure 3:**
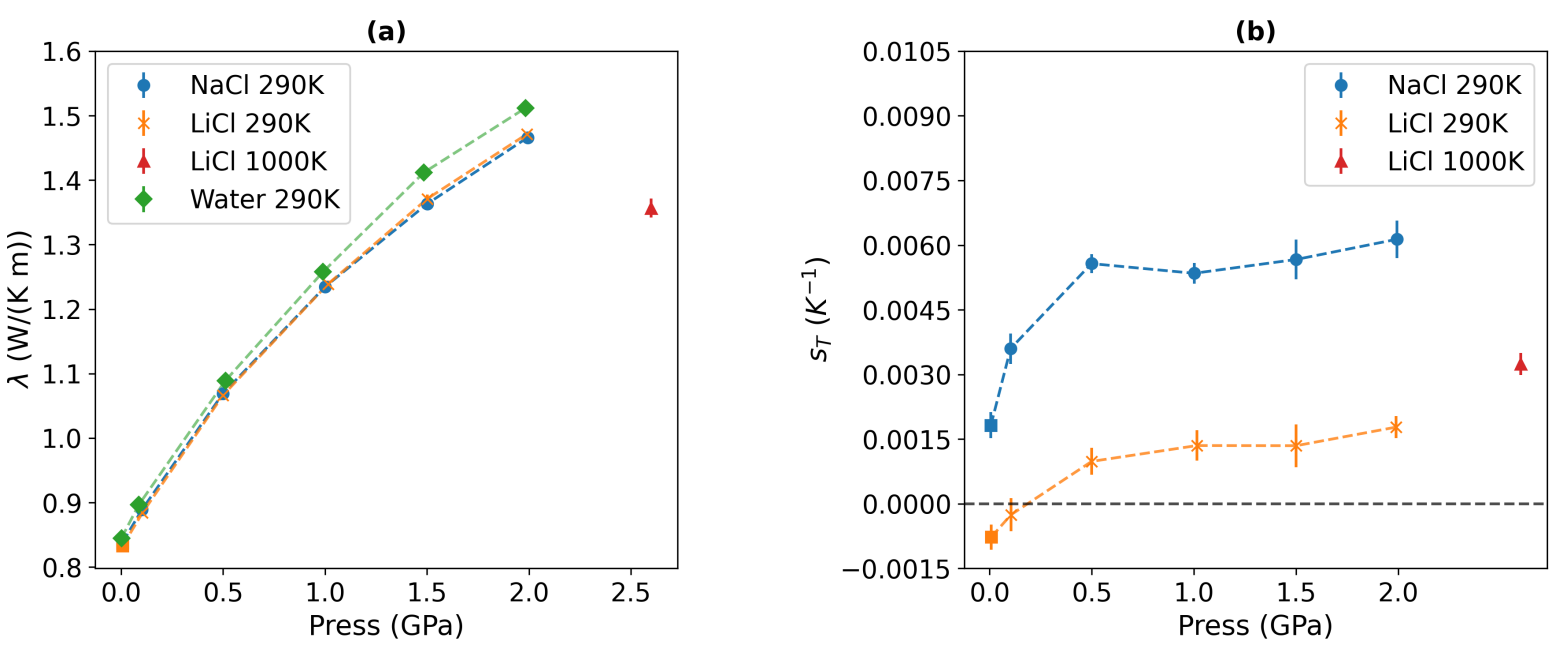
(**a**) Thermal conductivities and (**b**) Soret coefficients at 290 K temperature, 10^−4^, 0.1, 0.5, 1, 1.5, and 2 GPa pressures for 1 m NaCl and LiCl solutions. The square represents the thermal conductivity at 298 K and 31 bar reported in Ref. [21]. The green points in (**a**) represent our thermal conductivity data for pure water at 290 K temperature. The red triangles represent the thermal conductivity and Soret coefficient of 1 m LiCl solutions at 1000 K and 2.6 GPa pressure. The numerical data are compiled in Table A2.

Figure 3a shows the thermal conductivities of 1 m NaCl and LiCl solutions as a function of pressure for the 290 K isotherm. Our previous simulations demonstrated that the thermal conductivity converges within 10–30 ns at temperatures above 220 K for TIP4P/2005 water at various isobars, ranging from 1 to 1200 bar [43]. We followed this approach to obtain running averages during the production simulation phase.

The thermal conductivity of LiCl and NaCl solutions increases with pressure. We do not find a significant difference in the thermal conductivity of these solutions. This result agrees with previous studies of these solutions at near-standard conditions [21]. In that study, it was shown that the thermal conductivity of alkali halide solutions features a stronger dependence on the anion mass and a weaker dependence on the cation type. The thermal conductivity of the electrolyte solutions is lower than that of pure water (see Figure 3). We observe a 1–3% reduction in conductivity for 1 m NaCl and LiCl solutions, and the reduction increases with rising pressure. The reduction in the thermal conductivity in solutions relative to pure water is consistent with experimental and computer simulation studies at standard conditions [21,56]. Previous investigations [21] have demonstrated the mechanism behind the decrease in thermal conductivity upon adding salts: a decrease in the thermal conductivity correlates with an increase in the molar volume of the solution. However, the speed of sound varies depending on the type of salt. The reduction in thermal conductivity is also correlated with a disruption of the hydrogen bond structure of water, which is stronger at higher salt concentrations.

Advancing the discussion below, we note that water’s characteristic tetrahedral structure is considerably disrupted at high pressures, >0.1 GPa. Therefore, the high thermal conductivity observed in the high-pressure range is unrelated to water’s hydrogen bonding network.

**Figure 4:**
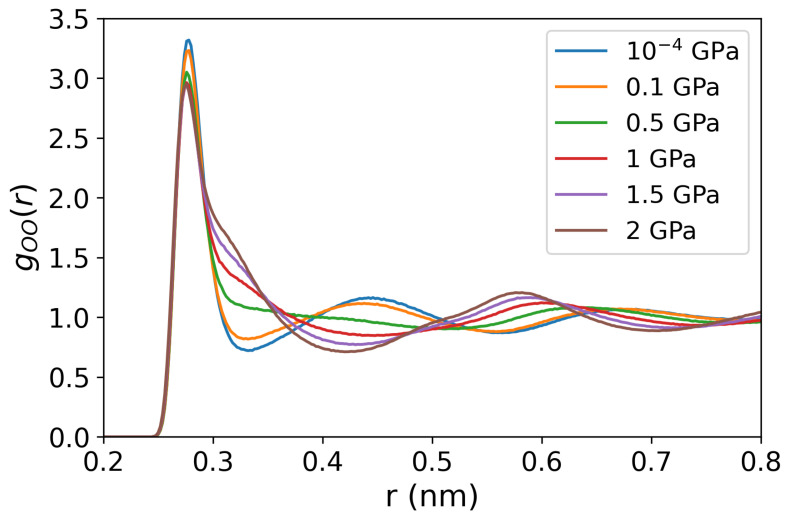
Oxygen-oxygen radial distribution functions of pure water at 290 K and various pressures (10^−4^, 0.1, 0.5, 1, 1.5, and 2 GPa).

**Figure 5:**
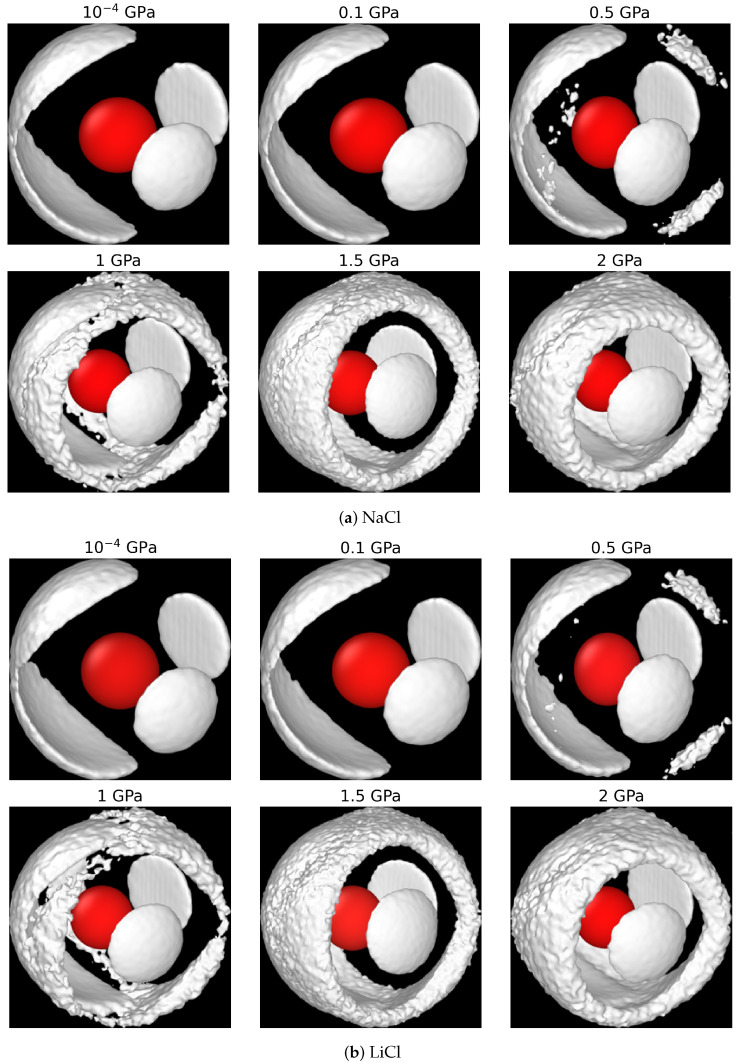
Spatial distribution functions $g_{OO}\left(r,\Omega \right)$ for the 1 m NaCl (**a**) and LiCl (**b**) solutions at 290 K and $10^{-4}$, 0.1, 0.5, 1, 1.5, and 2 GPa pressures. The central oxygen, colored in red, is shown for reference. Image generated with OVITO [58].

Our Soret coefficients are shown in Figure 3b. The Soret coefficients of both chloride solutions increase with pressure up to 0.5 GPa, reaching a plateau at higher pressures, where the Soret coefficient becomes nearly independent of pressure. At these elevated pressures, water loses the typical tetrahedral order (see Figure 4, and the radial distributions resemble those observed in simple liquids. The analysis of the spatial distribution function of water (see Figure 5) also supports this notion.

We find that the solutions become more thermophobic under pressure. This effect is particularly evident in the ${\mathrm{Li}}^{+}$ salt, whose Soret coefficient exhibits a sign change at *P*∼0.186 GPa. At this pressure, the solution transitions from thermophilic to thermophobic. This transition correlates with the systematic loss of tetrahedral order, as indicated in our radial distribution functions (see Figure 4). The impact of the pressure is also reflected in the self-diffusion coefficient, which we calculated from equilibrium simulations (see Table A3) and the results obtained at standard conditions are consistent with the experimental data [57]. The self-diffusion coefficients of both cations and anions remain nearly constant at pressures below 0.5 Gpa, but exhibit a sharp decreasing trend upon compression, decreasing to approximately half of their initial value from 0.5 to 2 GPa.

Recently, Zhang et al. investigated pure water under extreme conditions using neural network (NN) potentials, targeting pressures up to 22 GPa and temperatures in the 1000–2000 K range [11]. These conditions are found in the Earth’s mantle. The authors concluded that the thermal conductivity under these extreme conditions exhibits slight dependence on temperature while increasing with pressure [11]. Understanding the properties of ionic solutions at high pressure (approximately 1 GPa) and temperature (approximately 1000 K) is crucial for modelling deep fluids [37]. Therefore, we conducted additional NEMD simulations of 1 m LiCl solutions at high temperatures (1000 K) and extreme pressures (2.6 GPa) (see Figure 3). The density predicted with the TIP4P/2005 model for the 1 m LiCl solution (1.17 ± 0.003 g/cm^3^) is comparable to the NN potential predictions for pure water (1.20 g/cm^3^) [11]. Our thermal conductivity for the LiCl solution is 1.36 ± 0.02 W/(K m), a value similar to that reported under the same thermodynamic conditions for pure water modelled with the NN potential at 1.14 ± 0.22. The Soret coefficient under extreme conditions is more thermophobic than that of the LiCl solutions at 290 K in the 0.1–2 GPa range. This observation supports our perspective that disrupting the characteristic hydrogen bond structure of water (through increased temperature and pressure) enhances the thermophobicity of the solutions. Our results demonstrate that thermodiffusion is a significant coupling process under conditions encountered in the Earth’s mantle.

**Figure A2:**
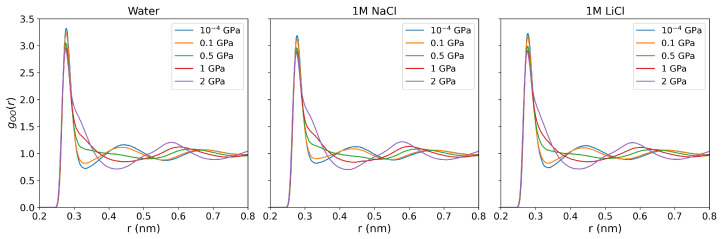
Oxygen-oxygen radial distribution functions for pure water, 1 m NaCl, and LiCl solutions at 290 K and various pressures (10^−4^, 0.1, 0.5, 1, and 2 GPa).

We now discuss the structural changes the solutions undergo upon increasing the pressure. We show in Figure 4 the oxygen-oxygen RDFs for pure water at 290 K temperature and pressures from 10^−4^ to 2 GPa. A considerable disruption of the water structure is observed at pressures higher than 0.5 GPa, evinced by the shift of the second intermolecular peak around r=0.45 nm to longer inter-molecular distances at 0.5 GPa pressure and above, the second-highest peak is located at ∼$2\;r_{max}$, where $r_{max}$ denotes the location of the main maximum in the radial distribution function, consistent with the structure for a simple liquid. The O-O RDFs of NaCl and LiCl solutions show similar behavior, including the peak positions and values in response to rising pressures (see Figure A2 in the Appendix A).

The SDFs of the aqueous solutions in Figure 5 further support the observations reported above. A significant loss of local tetrahedral order in water is observed at 0.5 GPa, and the tetrahedral order completely disappears at higher pressures. This observation applies to both chloride solutions, LiCl and NaCl. The liquid structures at 1.5 and 2 GPa pressures closely resemble a simple liquid, agreeing with the findings of pure water at extreme conditions in Ref. [9], and the SDFs reported in analyses of X-ray scattering of aqueous solutions at high pressure [33].

**Figure 6:**
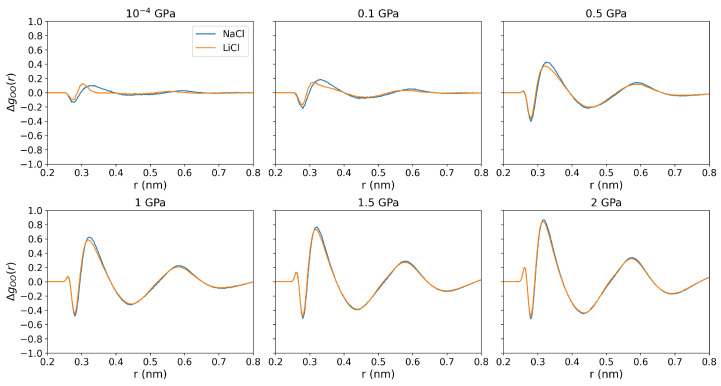
Perturbation of the oxygen-oxygen radial distribution function in 1 m NaCl and LiCl solutions at 290 K and pressures of 10^−4^, 0.1, 0.5, 1, 1.5, and 2 GPa. The different RDFs are presented using the O-O RDFs of water at standard pressure, i.e., $\Delta g_{OO}\left(r\right)=g_{OO,solution}\left(r\right)-g_{OO,purewater\;(10^{-4}\;GPa)}\left(r\right)$.

We computed the difference between the O-O RDFs in the electrolyte solutions and pure water at standard pressure to investigate how ion-water interactions vary at elevated pressures (see Figure 6). The notable change at around the second peak (0.4–0.5 nm) as pressure goes up is associated with the loss of tetrahedral structure, and the disruption increases with rising pressure. At low pressures (10^−4^ and 0.1 GPa), LiCl has a small impact on the O-O RDF, mainly around the region near the first coordination shell, whereas NaCl induces more significant changes at distances 0.35-0.5 nm. As pressure increases, the disruption of the O-O structure in the 0.35–0.5 nm range rises significantly, At 0.5 GPa and above, the water hydrogen bond orientational structure disappears (see Figure 6), and the observed changes are independent of the type of cation, ${\mathrm{Li}}^{+}$ or ${\mathrm{Na}}^{+}$. Overall, we confirm a correlation between thermophobicity and the loss of tetrahedral structure, particularly in the 0.35–0.5 nm range. This observation is consistent with the conclusions in reference [21] for chloride solutions at standard pressure.

**Figure 7:**
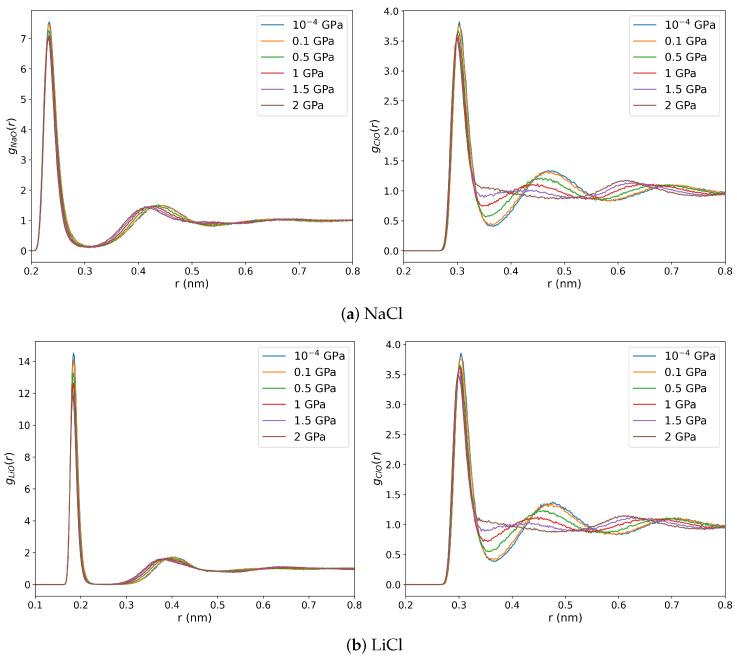
Oxygen-ion radial distribution functions for 1 m NaCl (**a**) and LiCl (**b**) solutions at 290 K and 10^−4^, 0.1, 0.5, 1, 1.5, and 2 GPa pressures. The left and right panels show the oxygen-cation and oxygen-anion RDFs, respectively.

We show the ion-oxygen RDFs in Figure 7. The coordination numbers obtained by integrating the radial distribution function up to its first minimum are reported in Table A1. The pressure has a minor impact on the cation-water structure, especially for ${\mathrm{Li}}^{+}$, where the Li-O coordination number equals 4 for all the isobars we studied. In contrast, the Cl-O RDFs are significantly influenced by compression. The second Cl-O peak decays from 0.1 GPa onwards and disappears above 1.5 GPa. These changes in the RDF indicate a systematic loss of a well-defined solvation structure, and the Cl-O coordination number increases significantly up to ∼20 at ∼2 GPa. This large increase in chloride coordination number (and shift of the main Cl-O peak to longer distances) agrees with the data reported in previous X-ray diffraction measurements, where an increase from 6 to 16 was reported at 1.7 GPa pressure and 300 K [33]. Similarly, the small impact of pressure on the cation-coordination number observed here is consistent with those X-ray diffraction measurements. The disruption of the hydration shell around the anions at these high pressures is linked to the drastic alteration in the hydrogen bonding network. Significant effects in the anion hydration were also reported in x-ray absorption studies of RbBr at 2.8 GPa pressure, along with a concomitant loss of orientation order in the hydration shells [59].

**Figure A3:**
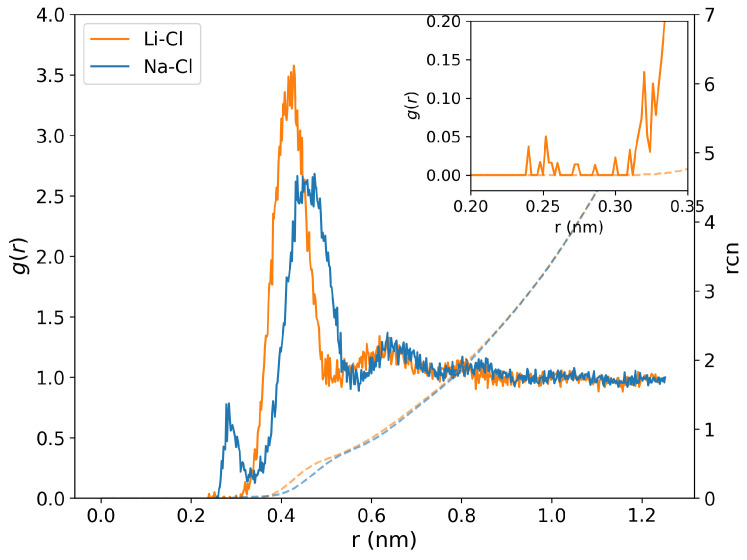
Sodium-chloride (blue curve) and lithium-chloride (orange curve) radial distribution functions and corresponding running coordination numbers (dashed line) for NaCl and LiCl solutions at 290 K temperature and 2 GPa pressure. The inset represents the zoomed-in region for the radial distance between 0.2 and 0.35 nm, providing a closer view of the first minimum in the radial distribution functions for Li-Cl.

We performed additional analyses of the ion-ion correlations to quantify the ion association. Figure A3 in the Appendix A shows the cation-anion RDFs and corresponding running coordination numbers at 2 GPa for the NaCl and LiCl solutions. The radial distances for the first minimum in Na-Cl and Li-Cl RDFs are ∼0.28 and 0.33 nm, respectively, consistent with the positions found in previous simulation studies [20,27,28]. Sakuma and Ichiki simulated NaCl solutions in the 670–2000 K temperature range and up to 2 GPa pressure, and they found that the cation-anion coordination numbers tend to decrease with increasing pressure and decreasing temperature [27,28]. At 290 K and 2 GPa, we do not find evidence of significant ion pair formation, particularly for the LiCl solutions (see Figure A3 in the Appendix A).

**Figure 8:**
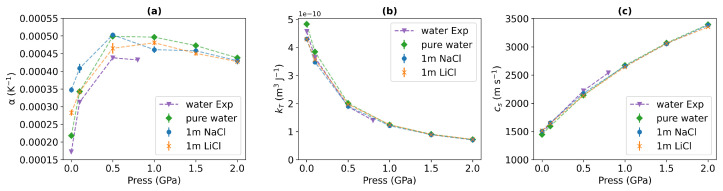
(**a**) Isobaric thermal expansion coefficient, (**b**) isothermal compressibility, and (**c**) speed of sound of pure water and 1 m NaCl, and 1 m LiCl solutions at 290 K. The data correspond to the following pressures: 10^−4^, 0.1, 0.5, 1, 1.5, and 2 GPa. The purple triangles represent the experimental data from Ref. [60] at 10^−4^, 0.1, 0.5, and 0.8 GPa pressures and 290 K.

Finally, we explore the dependence of the response functions—isobaric thermal expansion and isothermal compressibility on pressure. Figure 8 displays these properties for pure water and the aqueous solutions. Moreover, we have included experimental data for the isobaric thermal expansion and isothermal compressibility of water. These properties were extracted from the equation of state reported in Ref. [60]. Figure 8 shows small differences between the data for pure water and the aqueous solutions. For both the simulated and experimental isobaric thermal expansion coefficient (Figure 8a), all the liquids exhibit a local maximum at around 0.5 GPa. This pressure corresponds to the same range where we observe a significant disruption of the tetrahedral orientational order (see Figure 5). As the pressure increases, the thermal expansion coefficient difference between water and ionic solutions diminishes. The simulated thermal expansion coefficients are 15% higher than the experimental data on average. This deviation is similar to the ∼9% overestimation observed at 298 K and 1 bar with TIP4P/2005 [39].

Our simulation results align closely with the experimental isothermal compressibility and speed of sound data, confirming the accuracy of the TIP4P/2005 model. The isothermal compressibility (see Figure 8b) decreases in the whole pressure range, and the speed of sound (see Figure 8c) increases with rising pressures. The thermal conductivity is correlated with the speed of sound via the Bridgmann equation [61]. While this equation is not always accurate, it contains the key ingredients to understand qualitatively the dependence of thermal conductivity on the speed of sound. The Bridgman equation has inspired a recent formulation where the speed of sound appears explicitly as a key variable [11]. Moreover, the lack of dependence of the speed of sound on the cation type is consistent with the similar thermal conductivities of NaCl and LiCl (see Figure 3a).

## 4. Conclusions

We have examined, using non-equilibrium molecular dynamics and state-of-the-art rigid empirical force fields, the thermal transport properties and thermodiffusion of two alkali halide solutions, 1 m NaCl and 1 m LiCl, under GPa pressures. These pressure conditions are significant for crustal fluids.

The thermal conductivity of the solutions does not change substantially with the type of cation. In line with experiments and previous simulations, adding LiCl and NaCl diminishes the thermal conductivity of water, with the reduction being more pronounced (approximately 3%) at GPa pressures. The thermal conductivity varies considerably with pressure, increasing nearly twofold compared to pure water at standard conditions. This increase correlates with the substantial reduction of the solution compressibility as pressure rises, along with the accompanying increase in the speed of sound. This correlation is clearly observed when comparing the data in Figure 3a and Figure 8. Therefore, the solutions and pure water at GPa pressure are efficient heat conductors.

The Soret coefficients of LiCl and NaCl increase with pressure, reaching a plateau above 0.5 GPa. The thermophobicity of the solutions is closely linked to the loss of tetrahedral order and hydrogen bonding in water. At high pressure (approximately 0.5 GPa), the water structure resembles a simple liquid. The hydration structure of ${\mathrm{Li}}^{+}$ does not significantly change with pressure, and the tetrahedral water coordination of ${\mathrm{Li}}^{+}$ is maintained up to 20 GPa. This result indicates that the thermophobicity of the alkali halide solutions observed at GPa pressures is associated with the loss of the hydrogen bond structure in water, highlighting the role of hydrogen bonds in determining the transition from thermophilic to thermophobic states. This correlation aligns with previous conclusions regarding thermodiffusion processes under standard conditions [16]. Besides, This type of behavior has also been observed in concentrated aqueous solutions [21], and it is noted here when we apply high pressures, where water loses its orientational order (see Figure 8). This led us to conclude that thermophilicity is favored in situations with weak hydrogen bonding, while strong hydrogen bonding leads to thermophilicity, and ions are excluded from the colder regions in that case. Based on this, we expect that thermal gradients will lead to salt migration towards cooler regions in crustal fluids.

The simulations reported in this work were performed with rigid water models. Bond and angular degrees of freedom might be relevant in extreme conditions occurring in the earth’s mantle, and these effects might be included. Previous simulations of the TIP4P/2005 flexible model reported slightly higher thermal conductivities than the rigid model at 300 K and standard pressure [62]. Machine learning potentials and the introduction of quantum corrections [63] might also provide a focus for future investigations.

## Data Availability

The original contributions presented in this study are included in the article/Appendix A. Further inquiries can be directed to the corresponding author.

## Abbreviations

The following abbreviations are used in this manuscript:

NEMD	Non-equilibrium Molecular Dynamics
PPPM	Particle-Particle Particle-Mesh
NPT	Isothermal-isobaric
RDF	Radial Distribution Function
SDF	Spatial Distribution Function
**Symbols**	
*A*	Cross-sectional area of simulation box
*b*	Solute molality
$c_{s}$	Speed of sound
$C_{P}$	Isobaric heat capacity
$C_{V}$	Isochoric heat capacity
*D*	Inter-diffusion coefficient
$D_{T}$	Thermal diffusion coefficient
*g*	Ratio of the local particle density in the radial distribution function
*H*	Enthalpy
$J_{i}$	Mass flux
$J_{q}$	Heat flux
$k_{B}$	Boltzmann constant
$k_{T}$	Isothermal compressibility
$L_{x},L_{y},L_{z}$	Simulation box length in *x*, *y*, *z* dimensions
$r_{\max}$	Location of the main maximum in the radial distribution function
$s_{T}$	Soret coefficient
$T_{H},T_{C}$	Temperature of the hot and cold thermostatting regions
*V*	Volume
*w*	Weight fraction
*x*	Molar fraction
α	Isobaric thermal expansion coefficient
λ	Thermal conductivity
∇μ	Chemical potential gradient
∇T	Thermal gradient
ρ	Density
σ	Entropy production
$\dot{Q}$	Heat rate

## Appendix A

**Table A1:** Coordination numbers between the ions and oxygen in water, and the radial distance for the calculation of the coordination numbers: 1 m NaCl (**Top**) and LiCl (**Bottom**) solutions at 290 K and 10^−4^, 0.1, 0.5, 1, 1.5, and 2 GPa pressures.

NaCl Press (GPa)	$n_{Na-OW}$	$r_{Na-OW}\left(nm\right)$	$n_{Cl-OW}$	$r_{Cl-OW}\left(nm\right)$
10^−4^	5.55	0.32	5.94	0.37
0.1	5.64	0.32	5.98	0.36
0.5	5.84	0.31	6.05	0.35
1	5.95	0.31	6.20	0.35
1.5	6.03	0.31	23.03	0.52
2	6.06	0.30	19.87	0.49
LiCl Press (GPa)	$n_{Li-OW}$	$r_{Li-OW}\left(nm\right)$	$n_{Cl-OW}$	$r_{Cl-OW}\left(nm\right)$
10^−4^	4.00	0.27	5.81	0.36
0.1	4.00	0.26	5.99	0.37
0.5	4.00	0.25	6.15	0.36
1	4.00	0.26	6.55	0.35
1.5	4.00	0.24	22.66	0.51
2	4.00	0.24	18.38	0.47

**Table A2:** Results of the NEMD simulations. The pressures are in GPa, temperature in *K*, density (*ρ*) in g/cm^3^, thermal conductivity (*λ*) in W/(K m) and Soret coefficient ($s_{T}$) in the unit of $K^{-1}$. δλ and $\delta s_{T}$ are the standard deviations calculated from the replicas.

1 M NaCl						
Press	Temp	ρ	λ	δλ	$s_{T}$	$\delta s_{T}$
0.10	290.1	1.08	0.889	0.001	3.60 × 10^−4^	3.52 × 10^−4^
0.50	290.2	1.19	1.069	0.002	5.57 × 10^−3^	2.20 × 10^−4^
1.00	290.1	1.28	1.235	0.005	5.35 × 10^−3^	2.44 × 10^−4^
1.50	290.2	1.34	1.363	0.004	5.67 × 10^−3^	4.60 × 10^−4^
1.99	290.4	1.39	1.466	0.003	6.13 × 10^−3^	4.32 × 10^−4^
1 M LiCl						
Press	Temp	ρ	λ	δλ	$s_{T}$	$\delta s_{T}$
0.10	290.2	1.06	0.884	0.002	−2.55 × 10^−4^	3.80 × 10^−4^
0.50	290.1	1.17	1.066	0.002	9.82 × 10^−4^	3.10 × 10^−4^
1.01	290.1	1.26	1.239	0.005	1.35 × 10^−3^	3.54 × 10^−4^
1.50	290.0	1.33	1.371	0.006	1.35 × 10^−3^	4.96 × 10^−4^
1.99	290.2	1.37	1.472	0.003	1.78 × 10^−3^	2.55 × 10^−4^
2.60	999.9	1.17	1.357	0.002	3.24 × 10^−3^	2.60 × 10^−4^
Pure water						
Press	Temp	ρ	λ	δλ		
1.70 × 10^−3^	289.9	1.00	0.845	0.002		
0.87	289.6	1.04	0.897	0.002		
0.51	289.9	1.16	1.089	0.002		
0.99	290.0	1.25	1.258	0.006		
1.48	289.2	1.31	1.412	0.001		
1.98	289.9	1.36	1.512	0.003		

**Table A3:** Self diffusion coefficients of ions ($Na^{+}\left(D_{Na}\right)$, $Li^{+}\left(D_{Li}\right)$, and $Cl^{-}\left(D_{Cl}\right)$) and corresponding standard errors in the unit of 10^−10^ m^2^/s: 1 molal NaCl (**Top**) and LiCl (**Bottom**) solutions at 290 K and pressures of 10^−4^, 0.1, 0.5, 1, 1.5, and 2 GPa.

NaCl Press (GPa)	$D_{Na}$	$\delta D_{Na}$	$D_{Cl}$	$\delta D_{Cl}$
10^−4^	5.56	0.26	9.64	0.10
0.1	5.29	0.46	9.63	0.16
0.5	5.77	0.09	9.10	0.09
1	5.65	0.41	8.51	0.12
1.5	3.41	0.43	5.70	0.28
2	2.82	0.39	5.04	0.38
LiCl Press (GPa)	** $D_{Li}$ **	** $\delta D_{Li}$ **	** $D_{Cl}$ **	** $\delta D_{Cl}$ **
10^−4^	5.86	0.25	11.93	0.49
0.1	5.84	0.47	10.31	0.27
0.5	5.75	0.17	10.37	0.69
1	4.09	0.19	7.08	0.14
1.5	3.39	0.55	6.10	0.57
2	1.86	0.51	5.19	0.28
